# Supplementary material for: Are carbon emissions trading and green financial instruments synergistic? -Comprehensive quantitative research based on content analysis
Source: PLoS One. 2024 Mar 7;19(3):e0298601. doi: 10.1371/journal.pone.0298601 (PMC10919601; doi:10.1371/journal.pone.0298601)
Supplement: S1 Data — (ZIP) [file pone.0298601.s001.zip › Data/地级市Z_F工作报告.zip!/╔╜╬≈/┬└┴║╩╨/└┤╫╘╣1⁄2╓┌║┼í╛┬φ┐╦╩2╛▌═°í┐_2011.docx]

各位代表：

现在，我代表市人民政府向大会作工作报告，请予审议，并请市政协委员和其他列席人士提出意见。

一、“十一五”时期国民经济和社会发展回顾

“十一五”时期是我市历史上不平凡的五年。五年来，我们积极应对国际金融危机严重冲击，努力破解经济运行和社会发展中的深层次矛盾，在抢抓机遇中阔步前行，在迎接挑战中砥砺奋进，圆满完成了“十一五”规划确定的各项目标任务。

过去的五年,是市域经济发展加快、排位趋前、势头趋强的五年。全市地区生产总值从2005年的309.5亿元增加到2010年的845.5亿元，年均增长14%，由全省第8位跃升到第4位；财政总收入达到196.2亿元，是2005年的3.26倍，年均增长26.7%，由全省第6位攀升到第3位；一般预算收入达到73亿元，是2005年的3.91倍，年均增长31.35%；规模以上工业增加值达到552.2亿元，是2005年的3.92倍，年均增长20%；全社会固定资产投资完成435.8亿元，是2005年的2.8倍，年均增长23.2%；五年累计完成投资1704.23亿元，是“十五”时期的4.36倍；社会消费品零售总额达到222.5亿元，是2005年的2.65倍；银行业金融机构本外币存款余额达到1134.98亿元，是2005年的3.21倍。我们可以自豪地说，过去五年我市发展跃上了新的台阶，今天的吕梁已经站在了新的历史起点上！

过去的五年，是产业结构调整深入推进，经济发展方式明显转变的五年。三次产业结构由6.7：60：33.3调整为5.1∶69.6∶25.3。一般预算收入占财政总收入的比重从31%提高到37.2%，占GDP的比重从6%提高到8.6%。林果、小杂粮、畜牧等优质特色农业加快发展，综合生产能力不断提升。煤炭资源整合取得重大进展，产业集中度大幅提高。焦化、冶金等传统支柱产业质量效益明显改善，内部结构不断优化。煤化工、电力、铝镁、新型建材等接续产业发展迅速。科技成果产业化步伐不断加快，高新技术增加值占到地区生产总值的8.5%，全市新型工业化水平达到29.23％。第三产业加快发展，增加值由2005年的83.8亿元攀升到214.3亿元，年均增长14.9%。“两园经济”成为市域经济集约发展的生力军，近半数规模以上工业企业入驻园区，贡献率占到全市工业经济的40%；发展各类农业示范园区129个。节能环保与生态建设成效明显，连续开展五大环保攻坚战役，空气质量显著改善，市区污染指数从五年前的4.30下降到1.46，各县县城污染指数平均下降到1.94。全市重点河流地表水水质持续好转，集中式饮用水水源地水质全部达标。县县建成污水处理厂，全市污水日处理能力达到12.5万吨。万元GDP综合能耗累计下降22.38%，直接削减二氧化硫16万吨、化学需氧量1.7万吨，节能减排圆满完成“十一五”省定任务。新增造林面积284万亩，全市森林覆盖率和林木绿化率分别达到23.1%、35.9%，高于全国、全省平均水平。

过去的五年，是强农惠农力度不断加大，“三农”工作取得重大成效的五年。各级财政五年累计投入“三农”资金206.18亿元，是“十五”时期的3.07倍。农村生产生活条件不断改善，完成高标准旱作农田、节水灌溉农田、中低产田改造等耕地综合生产能力建设213万亩，治理水土流失面积8198.9平方公里，生态修复9494.8平方公里，新建淤地坝1041座。农业综合生产能力稳步提高，粮食产量达到10.4亿公斤，连续五年增产。深入实施“三大经济林覆盖”工程，累计发展干果经济林220万亩。大力实施农产品加工“513”工程，培育市级以上农业龙头企业72户，年加工能力达到80万吨，销售收入30.2亿元。增收渠道实现多元化，农民从二、三产业中所得收入占到总收入的70%以上。坚持集中财力办大事，在全国率先实行支农资金整合，累计整合资金35.71亿元。完成117个新农村试点村和906个重点推进村的建设任务，探索形成了富有特色的“十大创建模式”。大力实施整村推进、移民扶贫、产业扶贫、转移培训、教育扶贫等“五大工程”，54.9万绝对贫困人口解决了温饱问题。

过去的五年，是基础设施建设水平全面提升，城乡面貌发生显著变化的五年。全市国、省道干线公路改造和“通达工程”、“通畅工程”取得重大成果，汾柳、太佳、汾平高速公路建成通车，西纵高速、环城高速启动实施，境内高速公路总里程达到350公里，公路密度达到75.7公里/百平方公里，比五年前增加一倍还多。岢瓦铁路、太中银铁路竣工通车，孝柳铁路完成改造，吕临支线、山西中南部铁路大通道、太兴铁路建设顺利推进。吕梁民用机场全面开工。节水灌溉工程和提黄灌溉工程稳步推进，横泉水库、柏叶口水库、引文入川供水工程等一批骨干水源工程相继建成或开工。兴县500千伏输变电工程完成选址，中阳500千伏输变电工程及一批220千伏输变电工程建成投运，一批电源项目推进顺利，全市装机总容量达到216万千瓦，比2005年增加178万千瓦。“一市四区”规划编制全面启动，城乡规划体系日趋完善。全市累计投入城市基础设施建设资金217亿元，完成342个项目的新建和改造任务，城镇综合承载能力不断增强。城乡环境持续改善，吕梁市区连续四年荣获“省级卫生城市”，创建省级环境优美乡镇16个、省级生态村91个。

过去的五年，是各项改革深入推进，对外开放上档升级的五年。国有企业改革、国有资产监管体制改革取得积极进展。民营经济健康发展，民营企业增加值、纳税规模、从业人员分别占到全市总量的64.1%、60.3%、35.2%。市、县、乡政府机构改革顺利完成。深化财政体制改革，在全省率先建立企业信息平台。深化投资体制改革，规范招投标制度，政府投资效益进一步提高。集体林权制度改革深入开展，5个县完成主体改革。成功争取到全国土地综合整治规划编制试点政策支持。增值税转型全面实施。金融改革不断深入，孝义金岩、楼东和柳林联盛集团福龙公司等企业成功上市，32家小额贷款公司相继成立，4个村镇银行启动运行。招商引资成效明显，五年累计招引亿元以上项目37个，到位资金414.5亿元，实际利用外资10.6亿美元。与北京朝阳区缔结友好合作关系，成功举办一系列国家级体育赛事，一批精品文艺节目参加奥运会、世博会演出，扩大了对外影响力，展示了吕梁新形象。

过去的五年，是社会事业全面发展，人民生活水平不断提高的五年。各级财政社会事业支出累计完成500亿元，是“十五”时期的3.5倍，其中70%以上用于民生。教育“两基”目标圆满实现，初中和小学入学率分别达到94.2%和99.65%；建成省级示范高中10所；7个县（市、区）通过了省政府“科教兴县”示范县验收；吕梁学院挂牌成立，职业技术学院开工建设。科技研发资金连年增加，科技进步指数达到8.5%。人口自然增长率控制在5‰，人口计划生育工作连续八年保持全省先进。新型农村合作医疗在2008年就覆盖了全市13县（市、区），医疗卫生机构达标率达到90.4%。就业社保工作不断加强，全市就业总人数增加到176万人，社会保险综合覆盖率提高到86%。安全生产形势逐年好转，安全事故起数年均下降10%以上，亿元GDP死亡率累计下降72%。城市低保对象稳定在10.9万人，农村特困户保障对象达到18万人，基本实现了动态管理下的应保尽保。城镇居民人均可支配收入和农民人均纯收入分别达到15278元和3890元，年均增长13.46%和14.52%。城乡居民人均居住面积分别达到27.08平方米和28.99平方米，家用汽车、移动电话、互联网普及率显著提高。农村“五个全覆盖”目标圆满完成，居民出行、看病、上学、饮水、听广播、看电视更加方便。城乡公共体育设施加快完善，全民健身活动蔚然成风。全市文化广电、新闻出版、文物旅游、科普气象事业加快发展，青少年和妇女儿童事业、民族宗教、外事侨务、防震减灾、史志档案、老龄、残疾、人民武装等各项工作成绩显著。各项社会事业的大力推进，有效提高了全市人民的生活质量和幸福指数，促进了社会和谐。

过去的五年，是各级政府坚持依法行政，民主法治建设扎实推进的五年。各级政府自觉接受人大法律监督、政协民主监督和社会各界监督，认真听取和吸收各民主党派、工商联和无党派人士的参政议政良言，推进政府信息公开，决策科学化和民主化进程不断加快。市政府五年累计办理人大代表建议510件、政协委员提案2100件，办复率达到100%。积极推动“法治吕梁”建设，“五五”普法成效显著。全面落实政府系统党风廉政建设责任制，认真履行“一岗双责”，切实加强行政监察和审计监督，深入开展煤焦领域反腐败专项斗争，政风行风实现新的提升。社会治安综合治理继续加强，信访工作呈现“两好转、三下降”的良好态势。 各位代表，五年的风雨历程，谱写了吕梁发展史上的壮丽篇章，曾经以贫穷落后闻名于世的吕梁，已经成为生机勃发、受人瞩目的发展高地。大好局面来之不易。五年来，我们以科学发展观为统领，始终坚持把市委的领导作为做好政府工作的根本保证，始终坚持把人大和政协的监督作为推动政府工作的重要依托，始终坚持把依靠和凝聚全市人民力量作为实现转型跨越发展的强大动力，始终坚持把狠抓落实作为完成各项任务的有效举措，始终坚持把维护人民群众利益作为开展工作的出发点和落脚点。这是过去我们取得成绩的主要原因，也是未来再创佳绩的基本准则。在此，我代表市人民政府，向全市人民，向各级人大代表、政协委员，向各民主党派、工商联、无党派人士、各人民团体，向各位离退休老领导、老同志，向驻吕解放军指战员、武警官兵和公安干警，向所有关心支持吕梁建设的各界朋友，表示衷心的感谢和崇高的敬意！

各位代表，吕梁正处于转型跨越的攻坚期，正处于各种社会矛盾的叠加期，尽管这些年来我市总体面貌发生了很大变化，但是总量不足、质量不高、贫困人口多、发展不平衡仍然是现阶段的主要矛盾，特别是社会事业欠账多、百姓生活不殷实，发展方式粗放、生态环境脆弱，干部思想解放与政府职能转变的程度还不能适应转型跨越发展的要求等等，都是需要我们在今后工作中高度重视并加以解决的突出问题。对此，我们要进一步增强责任感和紧迫感，知难而进，奋力拼博，以更加坚定的信心、更加昂扬的斗志和更加扎实的工作，努力把政府各项工作做得更好，绝不辜负全市人民的期望和重托！

二、“十二五”时期经济社会发展的指导思想和目标任务



“十二五”是我市抢抓新一轮发展机遇，推动经济社会全面转型，实现整体跨越的关键时期。根据市委《关于制定国民经济和社会发展第十二个五年规划的建议》，我们编制了《吕梁市国民经济和社会发展第十二个五年规划纲要(草案)》，提交大会一并审议。

“十二五”时期我市经济社会发展的指导思想是：坚持以邓小平理论和“三个代表”重要思想为指导，深入贯彻落实科学发展观，以科学发展为主题，以加快转变经济发展方式为主线，以转型跨越发展为主旋律，以推进国家资源型经济转型配套改革试验区建设为统揽，更加注重资源型经济发展的质量和效益，更加注重经济结构调整，更加注重加快推进城镇化，更加注重安全生产和社会稳定，更加注重保障和改善民生，全面实施“五个转型、三大跨越”发展战略（简称“五三发展战略”），创造一个科学发展的“吕梁模式”和“吕梁速度”，到“十二五”末跨入全省经济社会发展强市行列。

转型发展的战略选择是：全面实施经济转型、城镇转型、生态转型、观念转型、管理转型，形成具有鲜明特色的“吕梁模式”。跨越发展的战略选择是：实现经济总量大跨越、固定资产投资大跨越、城乡居民收入大跨越，创造经济又好又快发展的“吕梁速度”。

“十二五”的主要目标和任务是：

（一）经济总量整体跨越。到2015年，全市地区生产总值达到1700亿元，年均增长15%；财政总收入达到400亿元，年均增长15%；全社会固定资产投资达到1500亿元，年均增长28%；城镇居民人均可支配收入和农民人均纯收入分别达到30000元和9000元，年均增长15%和18%。五项主要经济指标全部实现翻番。

（二）经济结构优化升级。坚持以安全生产为保障，以科技创新为支撑，以节能减排为手段，大力发展循环经济，推动吕梁经济由资源依赖型向创新驱动型转变，由资源型经济的产业结构向多元型经济的产业结构转型，逐步形成以特色农业为基础、优势工业为支撑、现代服务业为保障的三次产业协调发展的现代产业体系，力争到“十二五”末，高新技术增加值比重达到12%，服务业增加值年均增长19%，比重达到30%。

（三）“三农”工作不断加强。坚持因地制宜，错位发展，打造具有鲜明地方特色的县域经济，在发展“一村一品”、“一县一业”上实现新的突破。坚持“园区化”引领现代农业，加快构建两大核桃林带、三大农业片区、四大畜牧养殖基地、十大现代农业园区和十大农业旅游观光综合园区的农业产业化体系。全面推进社会主义新农村建设，继续完善农村路网、水电等基础设施，实施农村新的“五个全覆盖”，着力提高农民的社会保障水平、收入水平、健康水平和科技教育水平。把扶贫开发摆上更加突出的位置，提高扶贫标准，加大投入力度，围绕增加收入、增强能力、改善环境，实施扶贫开发工程，加快脱贫致富进程，力争提前一年实现新十年扶贫目标。

（四）城镇化进程全面提速。主动融入太原都市圈，加快建设“两个城镇带”，深入实施“大县城”战略，实现城市功能、品位、经济、管理及居民素质等要素与都市圈的深度对接。加快吕梁市区建设步伐，发挥区域中心城市辐射带动作用，建设吕梁医院、图书馆、展览馆、大剧院、工人文化宫、少年宫、文化广场、新闻广电大厦等标志性工程。引进市场机制，稳步推行市场化运作模式，最大限度吸引各类资本参与吕梁城镇化建设。到“十二五”末，全市城镇化率达到50%。强化基础支撑，加快“一港五网”建设步伐，构建吕梁空港以及公路网、铁路网、水网、电网、通讯网等基础支撑体系，实现飞机能上天、铁路通各县、高速全覆盖、供水网络化、电力高能化、通讯现代化的目标。

（五）生态环境明显改善。加强生态环境的保护与修复，大力开展造林绿化，着力打造“生态吕梁”，林木绿化率达到37.7%。继续推进节能减排，加快淘汰落后产能，持续开展重点行业和重点领域节能减排工程，支持节能技术和产品研发，从严控制污染物排放，形成比较完善的生态环境支撑体系。严格土地用途管理，着力提高单位土地的投资强度和产出率。推进煤层气、焦炉煤气、过境天然气和煤制天然气“四气合一”，建设“气化吕梁”。

（六）社会建设加快推进。实施科教兴市和人才强市战略，财政对科技、教育的投入增速高于一般性财政支出增速。优先发展教育事业，重视发展学前教育，巩固提高义务教育，加快普及高中教育，大力发展职业教育，全面推进素质教育，切实提高教育质量。大力支持科技创新，研发和引进一批关键技术。加快发展文化体育事业，扶持文化体育产业。深化医药卫生体制改革，健全完善医疗服务体系，重点提升乡镇卫生院服务水平，实现小病不出乡、大病不出县的目标。基本建立城乡社会保障体系，城镇基本社会保障覆盖率达到100%。继续强化社会就业工作，确保每个家庭至少有一名成员就业，城镇登记失业率低于4.5%。健全完善住房保障体系，加快保障性住房建设步伐，切实解决困难群体住房难题。调整收入分配秩序，着力缩小收入差距。大力加强安全生产，全面提高本质安全水平。

各位代表，展望“十二五”，我们面临着良好的发展机遇：我省成为国家资源型经济转型综合配套改革试验区，我市被列入全国土地综合整治规划编制市级试点，必将为吕梁转型跨越发展提供强有力的政策支撑；国家西部大开发、中部崛起和扩大内需战略的深入实施，必将为我市能源原材料产业发展及现代煤化工基地建设带来良好的市场环境；山西“一核一圈三群”市域城镇化格局的构建，必将为我市推动城镇化建设、促进城乡协调发展创造有利条件；“十一五”时期积累的建设成果和不断完善的发展环境，必将为市域经济又好又快发展奠定坚实基础；全市上下集中开展城市管理“三项整治”和干部纪律作风“三项整顿”，必将为我们进一步统一思想、凝心聚力，加快转型跨越发展提供有力保证。我们一定要正确把握形势，保持清醒头脑，积极主动作为，把良好的发展机遇转化为切实的前进动力。我们坚信，通过全市人民的共同努力，一个经济更加繁荣、社会更加和谐、人民更加幸福、山川更加秀美的新吕梁，一定会崛起在三晋大地！

三、2011年的工作



今年是“十二五”的开局之年。前不久，温家宝总理第三次来吕梁视察，对我市经济社会发展提出了新的要求。我们要深入贯彻落实温家宝总理视察吕梁重要讲话精神，突出“保投资、促转型、惠民生”的基本要求，大力推进以现代煤化工基地建设为重点的工业新型化，大力推进以特色农产品加工基地建设为重点的农业现代化，大力推进以“两个城镇带”建设为重点的市域城镇化，大力推进以“生态吕梁”建设为重点的城乡生态化，促进全市经济平稳较快发展、社会事业全面进步和民生进一步改善，努力在全省转型跨越发展中走在前列。

今年主要预期目标是：地区生产总值增长13%；财政总收入增长15%，其中一般预算收入增长15%；全社会固定资产投资增长24%；社会消费品零售总额增长19%；城镇居民人均可支配收入增长10.5%；农民人均纯收入增长12.5%；居民消费价格总水平涨幅控制在4%左右。

约束性指标是：万元生产总值综合能耗下降3.5%，万元工业增加值用水量下降4%，二氧化硫、化学需氧量排放量分别下降1.5%和2%，氮氧化物、氨氮和烟尘、工业粉尘排放量分别下降1.5%、1.5%、2%、2%。这些指标是硬性要求，必须不折不扣兑现落实。 实现上述目标，我们将重点抓好以下工作：

（一）千方百计保持经济平稳较快发展

全力实施项目牵动战略。重点建设煤炭提升、工业转型、农业龙头、三产牵动、基础设施惠民等五大类519个骨干项目，以项目建设保投资、促发展。全力抓好一批煤炭提升项目，重点加快西山煤电兴县晋兴能源3000万吨及一批千万吨级的大型煤炭集团建设步伐；抓好一批工业转型项目，培育壮大煤化工、焦化、冶金、电力、建材、装备制造以及煤层气综合利用等优势产业，重点推动山西金地“煤焦电化建”循环经济产业链、杏花村酒业集中发展区等项目取得实质性进展；抓好一批农业龙头项目，做大做强红枣、核桃、小杂粮等优势特色产业，在全省五大农业板块中凸显吕梁经济林和小杂粮板块；抓好一批三产牵动项目，加快发展装备制造业，建设矿用机械、化工设备、汽车贸易等商品集散地和现代物流园区，重点推进吕梁矿机城、聚富汽贸城、孝义沃尔玛超市商务区等项目建设。全力抓好省级重点工程建设，年度投资额至少达到290亿元。不断完善和创新项目推进机制，强化项目管理，做到承载主体、统计监测、管理制度、服务环境、质量监管“五个到位”。加快项目建设进度，做到以“目标倒逼进度、时间倒逼程序、督查倒逼落实”，并将项目建设纳入年度目标责任制考核范围，严格奖惩兑现。

努力拓宽投资渠道。按照国家宏观政策导向，做好项目筛选和申报工作，争取上级政府投资。完善政银企合作机制，引导金融机构支持地方经济建设，使更多的信贷资金留在吕梁。认真落实国务院新“36条”，进一步激活民间投资，重点引导退出煤炭行业的资金转向非煤产业或基础设施领域。强化外来投资主体管理，凡在我市有资金活动的企业，必须注册成立分支机构，成为独立的利润中心。集团化企业要至少发展一个非资源性产业项目。

倾力增强县域经济活力。扩大县级经济自主权，力争有更多的县列入全省“扩权强县”试点。在统筹布局生产要素的基础上，鼓励各县（市、区）依托资源禀赋、区位条件和产业基础，发挥比较优势，培育特色经济，走符合当地实际的特色发展之路。支持汾阳小杂粮、岚县马铃薯、孝义设施蔬菜、文水蛋鸡等全省“一县一业”基地县与178个“一村一品”专业村，进一步强化特色，打响品牌，做好示范。扶持区域特色明显的汾阳白酒、交城机械加工尽快实现集群化发展。支持柳林、孝义率先进入全省“十强县”行列，支持离石区向全省“五强区”迈进，支持其他县加快发展，力争进入全省县域经济发展争先进位十强行列。

大力发展民营经济。健全政策扶持体系，认真落实国务院《关于进一步促进中小企业发展的若干意见》，以及省委、省政府关于民营经济发展的一系列政策措施，不断完善包括政策、制度、效能等在内的综合服务与支持体系，营造民营经济依法平等使用生产要素、公平参与市场竞争、同等受到法律保护的运营环境。创新民营经济发展路径，降低准入门槛，放宽准入领域，学习借鉴汾酒集中区筹资方式，坚持“政府主导、市场推动、企业自愿”的原则，引领民营经济跳出传统产业，投资新兴产业，同时强化创新驱动，推进集群发展，努力走出一条绿色、循环、高端的转型跨越发展新路。

（二）牢牢把握政府宏观调控方向

促进区域协调发展。认真落实国家和省主体功能区规划，着力形成科学合理、各具特色、充满活力的区域经济发展格局。坚持市级整体统筹，合理规划产业，打破行政区划界限，促进优势互补和各种生产要素合理流动。鼓励平川四县（市）重点发展新兴产业，加快建设一批精细化工、装备制造、高新技术、现代服务、农产品加工等非资源型和产业链终端项目；支持山区各县以循环经济为基本路径，大力推进农副产品加工等劳动密集型项目，突出抓好沿黄现代煤化工基地建设，同时发挥农副产品富集优势，支持建设农产品加工项目，为群众增收提供保障；引导以市区为中心的中部县（区）重点培育现代服务业，不断加快区域性中心城市建设步伐。 

努力维护物价稳定。大力发展生产，保障主要农产品、基本生活必需品、重要生产资料供应。深入实施“菜篮子”工程，大力发展设施蔬菜，年内新增设施蔬菜种植面积2万亩。充分发挥价格调节基金的杠杆作用，完善价格补贴制度，建立健全社会救助、保障标准与物价上涨挂钩的联动机制，保障困难群众基本生活。加强市场监管力度，严肃查处欺行霸市、哄抬物价行为。推动房地产业健康发展，落实房价调控措施，加快保障性住房建设步伐，年内开工建设4644套，至少完成廉租住房1000套、公共租赁住房330套、城市棚户区改造500套的建设任务。通过以上措施的落实，力争全市物价涨幅不高于全国、全省平均水平，市区房价涨幅低于城镇居民收入增幅。

切实做好财税工作。加强税收征管，优化收入结构，加大地方小税与排污费、资源补偿费、两权收入、煤炭可持续发展基金等征收管理；完善税收管理考核体系，将一般预算收入占比、非煤产业税收增长列入年度目标责任制考核内容，着力增加地方可支配财力。规范和改善财政支出结构，财政对教育、科技、农业投入增幅高于经常性财政收入增幅；坚决压缩一般性支出，严控楼堂馆所建设，努力实现公务接待、会议、出国（境）三项费用零增长；严格执行人代会批准的财政预算，切实增强预算约束力。加强政府债务管理，规范政府举债融资行为。

不断完善基础设施。交通方面：全力推进吕梁民用机场建设。加快中南部铁路大通道、吕临支线、太兴铁路建设进度，年内争取开工建设配套战略装车站，做到路站同步建设、同步投运；启动阳双铁路交口支线前期工作，争取吕梁—太原城际快速铁路尽早开通。进一步完善公路网络，加快环城高速、西纵岢临高速吕梁段与临离高速等项目建设，力争西纵高速离交段年内开工，做好西纵高速石楼连接线选线前期工作；继续加大县乡公路、农村公路改造力度，加快完善矿区、林区、园区路网体系，抓好九条出市公路、三条旅游公路建设。电力方面：利用二氧化硫排污权有偿使用和交易试点政策机遇，争取一批重大电源项目获批开工，加快孝义500千伏输变电工程前期工作，开工建设兴县500千伏及一批220千伏输变电工程。水利方面：贯彻落实中央一号文件精神，紧紧抓住山西大水网建设机遇，全力实施兴水战略，切实抓好新水源工程建设，启动黄河碛口水利枢纽工程争取工作，加快柏叶口水库配套及龙门供水工程、千年水库、沿黄提灌等骨干水利工程推进步伐，构建吕梁水网体系。

（三）坚持不懈调整产业结构

改造提升传统产业。深入推进煤炭资源整合、煤矿兼并重组，关死关实淘汰矿井，加快改造在建矿井。批准开工建设的矿井，全面落实建设、施工、监理三方责任，全部建成标准化矿井。引导煤矿企业推进采洗一体化。严格控制焦化产能总量，置换提升现有产能，依托主焦煤资源和大型煤焦企业，加快焦化企业联合重组，提高产业集中度。积极推进岚县太钢500万吨特种钢项目，支持中钢和海威联合重组。大力淘汰落后水泥产能，积极发展三个1000万吨级水泥生产集中区，扶持华润水泥方山项目及福龙水泥等新型干法生产线项目建设。

培育壮大新兴产业。加快发展现代煤化工产业，重点在焦化副产品加工、煤气化和煤层气利用等方面取得新进展。大力发展材料加工业，重点培育碳纤维、陶瓷、高科稀土、新型墙体材料及镁铝合金、铝型材等资源型新材料。做大做优汾酒及红枣、核桃、小杂粮等特色农副产品加工业，鼓励发展光伏、煤层气清洁利用、LED生产制造、太阳能和风力发电等新能源产业。落实服务业发展政策措施，推进信息服务、中介服务、特色文化会展服务、商贸物流服务、社区综合服务等各类服务业协调发展，服务业增加值增幅达到12%以上。落实《吕梁市旅游发展总体规划》，重点建设和开发碛口风景名胜区、苍儿会生态旅游经济区、庞泉沟及红柳湾水上漂流等景区景点，打造精品线路，开发特色文化，旅游总收入增幅达到25%以上。

大力发展循环经济。全面对接《山西省循环经济总体规划》，进一步强化对循环经济发展的政策支持与资金投入。重点发挥园区的辐射带动作用，加大交城、文水、孝义3个省级经济开发区的改造提升力度，加快现代煤化工基地、太钢岚县钢铁基地、兴县氧化铝基地、孝义铝工业园、柳林主焦煤循环开发试验区、离石新型煤化工产业基地的推进步伐，努力实现土地集约利用、废物交换利用、能量梯级利用、废水循环利用和污染物集中处理,逐步形成企业小循环、园区中循环、区域大循环的格局。

各位代表，依托得天独厚的资源禀赋，我们已经奠定了坚实的发展基础；顺应经济发展的大趋势和转型跨越的新要求，现在吕梁经济整体上进入了脱胎换骨、浴火重生的关键时期。只要我们以坚韧不拔的毅力和壮士断腕的气魄，敢于创新，勇于舍弃，抓项目保投资，抓调控促统筹，抓转型谋长远，就一定能够保持市域经济平稳较快发展的良好势头，就一定能够走出一条“以煤为基、多元发展”的全新路径，就一定能够创造兴于煤而不困于煤、立足资源又超越资源的科学模式，就一定能够开辟又好又快发展的崭新局面！

（四）“四轮驱动”推进“三农”工作

完善强农惠农政策。认真落实国家和省委、省政府各项强农惠农政策，完善吕梁特色的“以工补农、以煤补农”机制，切实增加农业生产性投入，做到财政支出重点向农业农村倾斜，确保总量、增量逐年提高；基本建设投资重点用于农业农村基础设施建设，确保总量和比重进一步提高。坚持自愿和协商的原则，在土地流转、规模经营过程中保留农民工的承包田和宅基地，维护其合法权益。

培育现代产业。突出抓好玉米丰产方建设，带动全市玉米种植面积稳定达到210万亩以上。继续推进东西两山优质小杂粮产业开发，着力打造特色农产品生产加工基地。扎实推进农业服务体系建设，加快农技农机推广力度，提高农业生产经营组织化程度。继续完善农业标准化生产体系，逐步出台切合吕梁实际的农副产品市场标准化管理办法，确保让全市人民吃上放心蔬菜，努力使吕梁农副产品走向全国。切实抓好经济林建设，按照“稳定红枣、扩大核桃”的思路，完成50万亩核桃林栽植任务。全面对接省十大强农工程，深入实施农产品加工龙头企业“513”工程，筹备组建北京农产品直销中心，切实抓好面向大中城市的小型连锁专卖和“农超对接”，支持农业龙头企业做大做强，力争年内新增2个销售收入亿元以上的龙头企业,全市龙头企业销售收入达到35亿元。扶持规模健康养殖业加快发展，年内新发展规模养殖小区200个。

加强农业基础建设。重点实施耕地综合生产能力建设、中低产田改造、旱作节水农业“三大工程”， 年内完成中低产田改造31万亩、测土配方施肥170万亩、秸秆还田90万亩、水土流失治理40万亩。强化耕地保护和节约用地，加大土地开发整理和复垦力度，切实做好耕地保护工作。大力发展农业园区，重点扶持离石吴城农业综合园区、文水胡兰综合园区等十大现代农业园区和柳林联盛生态文化园、汾阳贾家庄农业生态观光园等十大农业旅游观光综合园区建设。

加快新农村建设。继续完善项目整合、资金捆绑、企业帮建、单位帮扶四项机制，突出抓好新确定的221个新农村重点推进村建设，确保完成“四化四改”和“五个一工程”年度目标任务。深入推进新农村建设“百村行动计划”，年内建成100个高标准新农村，并力争实现集中连片发展。

（五）统筹抓好城乡规划建设管理

优化城镇布局。加快构建“1273”四级城镇体系，重点推进“两个城镇带”建设。一是抓好太原都市圈特色城镇带建设，把握吕梁融入太原都市圈的机遇，承接产业转移，取得多赢成果。充分发挥平川四县（市）特别是孝义、汾阳两市的龙头带动作用，打造引领全市转型发展的引擎。二是抓好山上县（区）特色城镇带建设，加快“一市四区”各类规划编制及基础设施建设步伐，重点推进吕梁市区火车站站前广场、晋绥大道、北城区路网改造、城市主次干道提升、高速东西出口改造等项目建设，完善城区集中供热、供气、污水垃圾处理、新水源建设、天然气接用等公共服务体系，构筑全市城镇化发展的核心区。积极实施“大县城”战略,加快其余各县县城扩容提质步伐，引导生产要素、优势资源向县城集中。按照“五十年不落后”的标准，每个县（市、区）至少完成一条样板街建设。三是选择杏花村等30个左右基础条件较好、发展潜力较大的建制镇，支持率先发展。力争全年市政公用设施投资额达到32亿元以上，城镇化率至少提高1.5个百分点。 提升城镇品位。依托地域文化品牌，深度发掘沿黄文化，重点推动以碛口古镇为代表的一批历史文化底蕴深厚的小城镇发展。抓好文化遗产资源的普查与整理，注重开发孝义皮影、中阳剪纸、临县秧歌、岚县面塑等非物质文化遗产的经济潜质，在艺术资源集中的城镇，通过展演、制作、销售，开发富有地方特色的文化一条街、文化精品小区，建设特色文化镇。

实施精细管理。坚持把环境卫生、街道装饰、交通秩序“三项整治”作为加强城市管理、提升城市品位的主要切入点，在巩固现有整治成果的基础上，进一步扩大整治范围，形成工作常态，做到城乡全覆盖、推进一体化。要十分注重创新推动“三项整治”工作的路径，实现由治标保洁转向工程建设，实施项目化管理，科学规划，扎实推进；实现由突击治理转向长效机制建设，抓好环卫、交警、广告牌匾管理三支队伍建设，推行末位淘汰，保持动态平衡；实现由制度建设转向品牌创建，年内县县创建省级卫生县城，离石、孝义、汾阳争创全国卫生城市，孝义市争创国家级园林城市。同时大力营造“三项整治”工作氛围，加强舆论引导，加大督查力度，总结先进经验，树立先进典型。认真做好全省“三项整治”现场会的筹备工作。强化城市建设和管理的成本意识，创新城建项目管理机制，严格规范招投标制度，对条件成熟的项目探索引入代建制，严格预算管理，确保工程质量。

（六）持之以恒推进生态建设

大力推进以节能降耗为核心的技术改造。突出抓好电力、钢铁、焦化、化工、建材、煤炭等重点领域、重点工程、重点企业节能工作，对年综合能耗5000吨标煤以上的单位全部纳入重点用能单位进行管理，严格执行高耗能产品能耗限额标准；大力推广节能环保技术，积极发展清洁生产，努力提高资源综合利用率。继续淘汰落后产能，严格控制高耗能产业、产品发展，为全市重点项目、优势企业、新型产业腾出发展空间；严格执行节能评估审查、环评审批等制度，坚决制止不符合国家产业政策、环保政策的项目上马，从源头上转变畸重的产业结构与粗放的发展模式。 深入实施“蓝天碧水扩容提质工程”。制定并严格落实氮氧化物、氨氮及烟尘、粉尘减排的工作措施及考核办法，确保完成年度任务。着力提高市区集中供热率，最大限度地减少煤烟污染，全面控制城市扬尘污染，有序推进市区公交车和出租车燃用天然气改造，抓好机动车尾气污染、建筑工地施工和城市噪音污染整治，推进城市生活垃圾处理场建设，加强对饮用水源地周边、工业园区等重点流域、重点区域的环境执法监管。狠抓乡村环境建设，积极推广微生物肥料，推进大中型沼气工程建设，有效减少农村面源污染。

继续加强生态环境修复与治理工作。全力实施六大林业建设工程，大力推广“一矿一企绿化一山一沟”造林模式，不断引深石楼县碳汇造林试点，打造全省样板工程。全年营造林合格面积达到49.63万亩，林木绿化率增加一个百分点以上。加快水土流失综合治理和生态修复步伐，重点推进三川河、汾河流域生态环境综合修复治理，确保流域水质达到功能区指标要求。完善农村土地管理制度，抓好土地整治与修复，建立完善土地流转市场，优化土地资源配置，切实提高土地利用率和产出率。

（七）全力以赴加强以保障和改善民生为重点的社会建设

推进各项社会事业协调发展。实施学前教育三年行动计划，全面铺开一个乡镇一所标准化幼儿园建设工程，学前三年毛入园率达到61%以上；抓好中小学布局调整等重点工作，推进义务教育标准化建设；推动高中阶段教育内涵式发展；整合市直中等职业教育资源，加快吕梁职业技术学院建设步伐；支持特殊教育、继续教育发展；加强师德建设，提升教师与管理人员素质，提高全市教育的整体质量。深入推进医药卫生体制五项重点改革，提升新型农村合作医疗保障水平，新农合财政补助标准提高到每人200元；落实基本药物零差率销售政策，今年在所有政府举办的基层医疗卫生机构和村卫生室实施国家基本药物制度；加强基层医疗卫生服务体系建设，促进基本公共卫生服务逐步均等化，人均基本公共卫生服务经费由15元提高到25元；推进公立医院改革试点工作；年内在孝义市率先实施县域医药卫生“六个一体化”综合改革试点；深入开展“办人民满意卫生、创人民满意医院”活动；加强中医药服务能力建设。大力发展文化事业，加快构建覆盖城乡的公共文化服务体系，加大文化精品创作和文化遗产保护开发力度，实施文化产业发展规划，满足人民群众多样化需求。实施科技创新与人才强市战略，加快科技成果向现实生产力转化，形成产学研紧密结合的技术创新体系；落实人才发展规划纲要，创新人才发展体制机制，加快形成培养、引进、留住、使用人才的智力支撑体系。继续稳定低生育水平，实施出生缺陷干预，人口自然增长率控制在7‰以下。切实保护妇女和未成年人权益。统筹抓好新闻出版、民族宗教、全民健身、外事侨务、史志档案、人民武装、拥军优属、防震减灾等工作。

实施民生提质工程。启动新的“五个全覆盖”工程，全面完成农村街巷硬化、农村便民连锁店、农村文化体育场所、农村职业教育免费、新型农村社会养老保险等“五个全覆盖”年度任务。启动兴村富民工程，创新扶贫开发机制，引导涉农企业按照“公司+基地+农户”的经营模式，发挥辐射作用，带动农民增收；开展万名干部下乡住村活动，因村制宜制订农民增收翻番计划，力争年内取得新成效。实施就业工程，重点解决城镇新增劳动力、农村转移劳动力和城镇就业困难人员的就业问题，年内新增城镇就业3.18万人，城镇登记失业率控制在4.2%以内。实施社会保障扩面工程，把农民工、灵活就业人员、非公有制经济组织等作为城镇参保扩面重点，基本解决未参保集体企业退休人员的养老保障问题，稳定解决好关闭破产企业退休人员和困难企业职工的基本医疗保障问题，将“老工伤”全部纳入工伤保险统筹管理，今年城镇职工基本养老保险、城镇基本医疗保险净增人数分别达到0.5万人、7.64万人以上；加快新型农村社会养老保险制度建设，两年实现全覆盖任务，今年覆盖率超过80%；提高社会保障标准，企业退休人员基本养老金每月人均增加140元，城镇居民基本医保财政补助标准人均增加80元，城市和农村居民最低生活保障人均每月增加25元、22元。实施社会救助工程，完善救助体系，提高救助标准，做好社会福利及慈善工作，探索建立农户生活用煤基本保障制度。

加强和创新社会管理。完善基层管理和服务体系，建立重大工程项目和重大政策制定的风险评估机制。健全应急管理体制，着力提高政府应对突发公共事件的能力和水平。畅通群众利益诉求渠道，进一步做好市县党政主要领导公开电话办理工作，从源头上防止不稳定因素发生。突出抓好流动人口、重点人群、重点区域的管理和服务，不断完善社会治安防控体系，严密防范和严厉打击各类违法犯罪活动。树立“民以食为天、食以安为先”的理念，切实做好食品安全工作，确保人民群众饮食安全。

扎实抓好安全生产。强化安全发展理念，严格落实省政府12个行业118条安全生产规定，进一步强化政府、企业安全生产的“两个主体”责任和部门监管责任，持续引深安全生产专项整治，重点抓好煤矿安全生产，统筹做好非煤矿山、尾矿库、淤地坝、道路交通、消防、危险化学品、特种设备、易燃易爆物品和公共场所安全管理，严厉打击私挖滥采和非法违法生产经营活动，促进全市安全生产形势由明显好转向稳定好转转变。

各位代表，群众利益无小事，民生事业重如山。改善人民群众生存条件，提高人民群众生活水平，是各级政府义不容辞的责任。在新的一年里，我们将把更多的精力、更大的财力投向民生领域，把各项社会事业发展好，把事关群众切身利益的问题解决好，为使全市人民群众的生活更加幸福美好而不懈努力！

（八）不断加快改革开放步伐

全面启动转型综改试验区建设。按照“解放思想、不等不靠、先行先试、重点突破”的总体要求，把握战略方向，科学谋划布局，走出一条具有吕梁特色的综改试验之路。坚持靠前站位，主动争取，力争在项目准入、土地供给、金融支撑、财税返还、节能减排等方面取得较大进展。按照“一市一个试点县”的要求，年内在综改试点县建设方面取得实质性突破，力争走在全省前列，同时培育一批循环经济试点园区和试点企业。

扎实推进各项改革。以实施全国土地综合整改规划编制试点为契机，实施城乡建设用地增减挂钩政策，探索创新矿业用地新模式，提高建设用地保障能力。深化财税体制改革，加快完善省直管县财政管理改革试点和“乡财县管”财政管理方式。稳步推进资源性产品价格和要素市场改革，促进资源保护和经济转型。深化国有企业改革，以“一退两置换”为核心，加大依法破产和兼并重组力度，打好小型国有企业改革攻坚战。深化集体林权制度改革，全面完成主体改革任务。深化文化体制改革，加快文艺院团改制，培育骨干文化企业，推进文化产业大发展。深化社会事业和公共服务领域改革，扎实推进非基本公共服务市场化。深化事业单位试点改革，年内基本完成清理规范和分类工作。深化乡镇机构改革，建立精干高效的管理体制和运行机制。

着力提高对外开放水平。鼓励外来资金投向高新技术、新能源产业、新材料加工、节能环保产业、基础设施、服务业和现代农业，着力强化“选商选资”的意识。加强区域交流和合作，积极探索区域产能置换、产业对接、资源共享等方面的合作。创新招商方式，鼓励企业以商招商、点对点招商，切实提高招商效率。实施品牌战略，培育一批知名品牌，提升经济品质，增强竞争能力。积极争取与国外城市缔结友好合作关系。认真做好第六届中博会参展筹备工作。

（九）切实加强政府自身建设

扎实推进行政管理体制改革，努力实现由经济主导型政府向公共服务型政府转变。牢固树立抓发展是政绩，抓管理也是政绩的理念，把社会建设和管理放在突出位置，增强工作的科学性和发展的协调性。深化审批制度改革，规范行政审批行为，强化便民服务意识，不断提升市县两级政务大厅便民服务功能。加强学习调研，提高履职能力，切实增强领导干部谋划发展、统筹协调、公共服务、社会管理、维护稳定的本领，努力为经济发展提供良好的政务环境，为市场主体提供方便快捷、贴心舒心的服务。

各级政府工作人员要真正把心思放在干事业上，把本领用在促发展上，把功夫下在抓落实上。坚持深入基层、深入群众，问政于民、问需于民、问计于民，提高决策水平。坚持干实事，求实效，多做打基础、管长远、利全局的工作，努力创造出经得起实践和人民检验的政绩。切实强化争先进位意识，积极争取国家级、省部级荣誉称号和考核奖项。把涉及群众“菜篮子”等各类生活所需的工作摆上政府的重要议事日程，千方百计把群众的生产生活安排好。

坚持把依法行政贯穿于政府决策、执行、监督的各个环节，促进严格、公正、文明执法。认真落实行政执法责任制和过错追究制，严格依法行政，规范权力运行。自觉执行市人大及其常委会的决议、决定，坚持重大事项报告制度。支持政协履行政治协商、民主监督、参政议政职能，广泛听取各民主党派、人民团体、工商联、无党派和各界人士的意见建议，全面提高依法行政水平。 全面落实政府系统党风廉政建设责任制，加强惩治和预防腐败体系建设。坚持用制度管权、靠制度管人、按制度办事，树立廉洁政府新形象。规范行政执法行为，严厉查处执法环节的不作为、乱作为和各种违规违纪行为，深入推进煤焦领域反腐败专项斗争，认真开展重点领域渎职侵权问题专项整治工作。深化政务公开，保障人民群众的知情权、参与权和监督权，确保公共权力在阳光下运行。进一步加强政府部门和公用服务行业的政风行风建设，坚决查处损害群众利益的行为。

各位代表，工作纪律与作风，体现政府形象，决定行政效率，关乎事业成败。市委确定今年为 “作风建设年”，全市政府系统要认真落实各项要求，切实抓好纪律作风“三项整顿”工作。要严肃整顿工作纪律，严肃整顿会议纪律，严肃整顿办事纪律，严肃整顿廉政纪律，以铁一般的纪律规范行政行为；要大兴认真务实之风，大兴立说立行之风，大兴团结奋斗之风，以良好的作风提升行政效能；要细化考核内容，优化考核方法，强化考核监督，注重结果运用，以科学的考核办法引导政府系统各级干部增强责任意识，合力推动发展，不断开创政府自身建设新局面，赢得全市人民的信赖和支持！

各位代表！经济繁荣、人民富足、社会和谐的宏伟蓝图即将绘就，吕梁人民一定会乘势而上、昂首阔步迈向新的征程。让我们在中共吕梁市委的坚强领导下，万众一心，开拓进取，大力弘扬“吕梁精神”，以只争朝夕的干劲和不甘人后的拼劲，为实现“十二五”的良好开局，为开创我市全面转型、整体跨越的崭新局面而努力奋斗！
